# Encoder-decoder models for chest X-ray report generation perform no better than unconditioned baselines

**DOI:** 10.1371/journal.pone.0259639

**Published:** 2021-11-29

**Authors:** Zaheer Babar, Twan van Laarhoven, Elena Marchiori

**Affiliations:** Institute of Computing and Information Sciences, Radboud University, Nijmegen, The Netherlands; Taipei Medical University, TAIWAN

## Abstract

High quality radiology reporting of chest X-ray images is of core importance for high-quality patient diagnosis and care. Automatically generated reports can assist radiologists by reducing their workload and even may prevent errors. Machine Learning (ML) models for this task take an X-ray image as input and output a sequence of words. In this work, we show that ML models for this task based on the popular encoder-decoder approach, like ‘Show, Attend and Tell’ (SA&T) have similar or worse performance than models that do not use the input image, called unconditioned baseline. An unconditioned model achieved diagnostic accuracy of 0.91 on the IU chest X-ray dataset, and significantly outperformed SA&T (0.877) and other popular ML models (*p*-value < 0.001). This unconditioned model also outperformed SA&T and similar ML methods on the BLEU-4 and METEOR metrics. Also, an unconditioned version of SA&T obtained by permuting the reports generated from images of the test set, achieved diagnostic accuracy of 0.862, comparable to that of SA&T (*p*-value ≥ 0.05).

## Introduction

**Fig 1:**
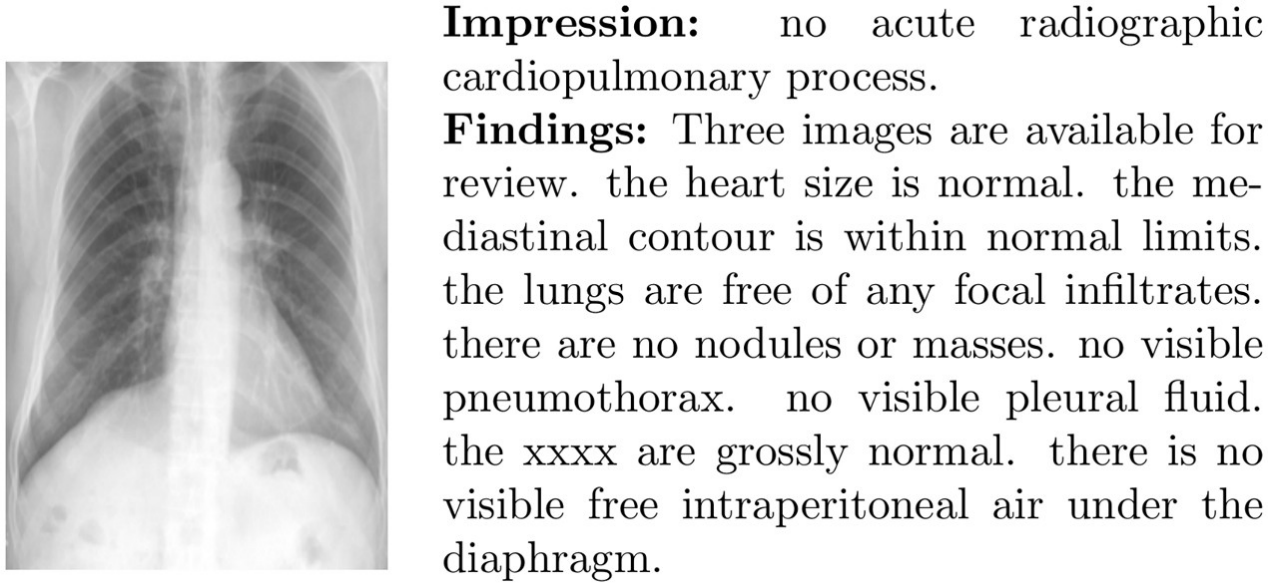
Example of impression and findings sections of a radiology report (Indiana U. Chest X-ray dataset). xxxx’s indicate removed keywords due to de-identification.

The written radiology report is an important means of communication between a radiologist and the referring clinician, and is crucial for patient care [1]. Radiologists produce reports with a standardized structure and clinical focus. Fig 1 shows the impression and findings sections of a typical radiology report of a chest X-ray image. The *impression* section is a single sentence summary, while *findings* describes technical observations about normal and abnormal conditions observed in the image, such as heart size and lung opacity, any abnormalities appearing at lungs, aortic and hilum, and potential diseases such as pneumothorax and consolidation [2].

Radiology reporting of chest X-ray images is time-consuming since it falls towards the end of the radiology workflow. Errors in radiological reports may results in incorrect clinical decisions. Methods to automatically generate reports from chest X-ray images can help radiologists and reduce their workload.

Recent advances in deep learning for image caption generation have also spurred research on ML methods for the automated generation of radiology reports. ML methods for this task mainly use the encoder-decoder approach: an encoder maps the input X-ray image to a latent space, which is used by a decoder to generate a sequence of words that forms a radiology report. This approach was originally introduced to generate captions of generic images [3, 4]. However, radiology reports differ from image captions in several ways: they are much longer, they have a highly standardized structure with large common parts, they contain observations about different parts of an X-ray image, and describe diagnostic content of the image in order to trigger the correct (re)action of the clinician. These characteristics make the problem of automatic radiology report generation more challenging, and it also makes it harder to evaluate generated radiology reports [5].

Image captioning methods are commonly assessed with metrics from the NLP literature, such as BLEU or ROUGE scores. These metrics are based on comparing the words in a generated caption against a reference. However, radiology reports have a rigid structure with many common phrases. So, two radiology reports with completely opposite meanings can still have a high score according to these metrics. Furthermore, the common phrases shared by radiology reports make it possible to find a single fixed report that, regardless of the contents of X-ray images, will have a high metric score when matched with the corresponding ground truth radiology reports. We call a method for making such a report an *unconditioned model*, because it does not depend on the input X-ray image, and we will show that unconditioned models can even outperform state-of-the-art encoder-decoder based ML methods. Next, we show that if we remove the dependency of an encoder-decoder model on the input, by inputting another unrelated X-ray image instead, the performance often does not degrade significantly.

Clearly, if the output of an automatic report generation method does not depend on the input image, then it is impossible for it to be clinical relevant. The fact that unconditioned models appear to perform well according to common text based metrics, while not being clinically relevant, casts serious doubts on the validity of these metrics. By extension, much of the literature that has compared methods by using these metrics should also be questioned. And we might wonder how good encoder-decoder based machine learning methods actually are on this task if they are outperformed by such baselines with respect to various assessment metrics.

There has been some effort to develop more clinically relevant metrics for automatically generated reports [2, 6–8]. But we will show that even with these metrics unconditioned models can outperform machine learning methods.

Overall, our investigation demonstrates that, in the context of automated report generation from chest X-ray images: 1) unconditioned models can outperform encoder-decoder ML methods with respect to various assessment metrics; 2) encoder-decoder models and their unconditioned version perform similarly; 3) tailored methods effectively exploiting the input X-ray image and specific characteristics of radiology reports need to be developed.

### Related work

Various machine learning methods to automatically generate radiology reports have been introduced, e.g., [2, 4, 6–11]. The main approach used by these methods is called the encoder-decoder architecture. The encoder-decoder architecture for image captioning was first introduced in the ‘Show and Tell’ method [3]. This method was later extended to ‘Show, Attend, and Tell’ (SA&T) [4], which adds an attention mechanism that focuses on different regions of the image for different parts of the generated caption. Later work has tried to adapt these encoder-decoder methods for radiology report generation. Notable is ‘Multimodal Recurrent Attention’ (MRA) [2], which generates a report one sentence at a time, and has independent modules for generating the impression and findings sections of a report. MRA first utilizes global image features to generate the impression part of the report, and then repeatedly takes the previous sentence and regional image features as input to generate the findings section, sentence by sentence. Recent focus has been on transformer architectures, which outperform recurrent neural networks on many tasks. A recent method based on this approach in the context of radiology report generation is ‘Conditioned Distil Generative Pre-trained Transformer 2’ (CDGPT2) [12]. CDGPT2 predicts tags for an image using CheXNet [13]. Furthermore, it extracts semantic features of predicted tags using pre-trained embeddings. Finally, it uses a pre-trained GPT2 language model conditioned on visual and semantic features to generate a radiology report.

Quality assessment of reports is mainly performed using traditional NLP validation measures, like BLEU [14], ROUGE [15], METEOR [16], and CIDEr [21]. Such measures are domain independent, hence they cannot be used to directly quantify the diagnostic quality of radiology reports, which is of core importance. As an extreme example, if the generated and ground truth reports differ only in a single semantically relevant word, like a negation, the generated report will have a very good BLEU score but a bad diagnostic content (opposite to that of the ground truth report). Other limitations of NLP metrics have been illustrated in [17], where a candidate caption semantically very similar to a reference one was considered, and the following observations were made: 1) all the metric scores decrease when some words are replaced with their synonyms, especially for CIDEr; 2) the metrics are not affected much by the introduction of additional (redundant) words in the sentences; 3) when the order of the words is changed, when *n*-grams with *n* > 1 are used, BLEU, ROUGE and CIDEr scores decrease notably, due to their dependence on n-gram matching.

Because of these issues, new validation metrics accounting for the diagnostic content of a radiology report have been proposed, e.g. [2, 6–8]. The metric introduced in [2], called Keyword Accuracy (KA), quantifies the diagnostic quality of a generated report by the fraction of relevant keywords it contains, based on a hand-made dictionary with keywords considered relevant. However, this metric still does not consider negation.

Another alternative approach is to extract diagnostically relevant labels from reports, and to compare the labels extracted from generated reports against those extracted from reference reports. This can be done by the CheXpert labeler, a publicly available https://github.com/stanfordmlgroup/chexpert-labeler NLP tool for extracting labels from radiology reports [18], which includes a rule-based classifier with 14 categories of diseases. This classifier is applied to the set of generated reports to compute validation scores, such as diagnostic accuracy, sensitivity and specificity. In [6–8] the CheXpert labeler was used to assess the diagnostic quality of reports generated by various baselines, including random selection of a report from the training set [6], and an unsupervised Recurrent Neural Network language model which generates free text without conditioning on input radiology images. Validation measures obtained from the CheXpert labeler have also drawbacks, since they depend on the specific method used to construct the rule-based classifier, and on the specific dataset used for its training.

## Materials and methods

### Data

We use two publicly available datasets: IU Chest X-Ray and MIMIC-CXR.

#### IU Chest X-Ray [19]

Contains 7470 X-ray images from 3955 patient reports. Each report contains one or more than one image and associated with a single patient. Each report consists of four sections: *impression*, *findings*, *comparison*, and *indication*. As in [2, 9], we use *impression* and *findings* together as the target report to be generated. In order to compare with results from the literature involving also clinical validation measures based on CheXpert, we also trained SA&T using only *findings* (we could not do this for MRA because this method explicitly uses also *impression*). We consider only records containing a pair of image views together with a complete textual report. After this, we are left with a total of 2775 records (each consisting of a pair of images and the associated medical report).

#### MIMIC-CXR [20]

To date the largest available collection of chest X-ray images along with textual reports. It contains 206,563 reports. Each report contains one or more chest X-ray images along with an associated textual report. Like in IU Chest X-Ray collection, each report in MIMIC- CXR also consists of four sections. As in [8], we use only *findings* section as the reference report. Therefore, we discard reports without *findings* section, hence are left with 155716 records.

We apply the following standard text cleaning and preprocessing steps to both datasets: 1) remove all non-alphabetic tokens 2) convert text to lowercase 3) apply tokenization. We retain stop words while do not apply either stemming or lemmatization. After preprocessing, we get 1933 and 12706 unique tokens for IU Chest X-ray and MIMIC-CXR, respectively.

### Encoder-decoder models

**Fig 2:**
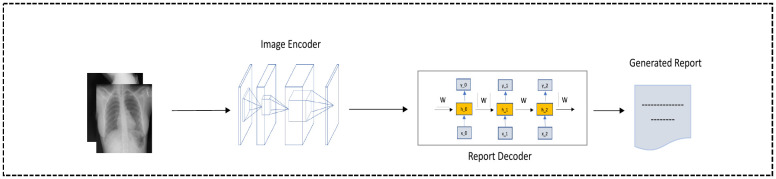
An encoder-decoder model. A CNN is used as encoder and an LSTM as decoder.

The main approach used by methods for (radiology) report generation is called the encoder-decoder architecture, illustrated in Fig 2: a deep neural network, typically a convolutional neural network (CNN), is used as encoder to extract features from an image, which are then used as input for a decoder, typically a Long Short Term Memory (LSTM) network, that generates a sequence of words. We conduct experiments with three algorithms based on encoder-decoder approach: **SA&T** [4] (our implementation https://github.com/zBabar/SA&T_NEW, https://github.com/yunjey/show-attend-and-tell), **MRA** [2] (publically available implementation suggested by the authors https://github.com/wangleihitcs/MedicalReportGeneration), and **CDGPT2** [12] (publically available authors’ implementation https://github.com/omar-mohamed/GPT2-Chest-X-Ray-Report-Generation).

### Unconditioned models

An unconditioned is a model whose output does not depend on the input image. Such a model forms a baseline for the performance of an automated radiology report generation system. If a method is truly extracting meaningful information from an X-ray image, it should outperform a baseline that does not depend on the input image at all.

Note that unconditioned models are not introduced with the aim to be used in practice, but only to show ineffectiveness of current state encoder-decoder methods for radiology report generation.

We will look at a simple class of such baseline models in particular, *single-report unconditioned models*, which always output the same single report for each input X-ray image. In order to define such models, we need some notation. Each report, generically denoted by *c*, is a sequence of words, which come from the set *W* of all considered words. Let W* denote the set of sequences of words constructed from *W*, forming a set

We use *X* = {*x*_1_, ⋯, *x*_*N*_} to denote a given set of *N* chest X-ray images and *R* = {*r*_1_, …, *r*_*N*_} the set of corresponding reference (ground truth) reports. concat(*r*, *r*′) denotes the concatenation of *r* with *r*′, and |*r*| denotes the length of the report (the number of words in *r*). The total length of all reference reports is denoted by $n_{R}=\sum_{i=1}^{N}\left|r_{i}\right|$.

An automated radiology report generation model takes as input an X-ray image and outputs a candidate radiology report. To train such a model, pairs (*x*, *r*) from a training set (*X*, *R*) are used.

If we know what evaluation metric *s* will be used to assess the quality of candidate reports generated by a model, then, in theory, we could construct the optimal single candidate report fitting the training set *R* as follows:

$$\begin{array}{c} c^{*}\left(s,R\right)=\underset{c\in C}{\text{arg}\;\text{max}}\;s\left(c,R\right). \end{array}$$ (1)

Where *C* is a set of reports, such that *R* ⊆ *C*, *s*(*c*, *R*) is the value of the evaluation metric when using report *c* as candidate for each X-ray image. Clearly, such a model is unconditioned.

In practice, computing $c_{s,R}^{*}$ is infeasible, because the number of reports to consider grows exponentially with the length of the report. Nevertheless, heuristic algorithms can be used. Here we consider two types of heuristics: a simple one that selects a best report in the training set (Baseline 1), and a more involved heuristic that greedily joins words into fragments and fragments into larger fragments, in order to optimize the BLEU-1 and BLEU-2 validation metrics (Baseline 2). These baselines are described below (A python implementation is publicly available at https://github.com/zBabar/metrics_based_validations).

**Baseline 1**. The first unconditioned baseline model simply outputs a report from the training set that maximizes a given metric *s*,

$$\begin{array}{c} c=\underset{r\in R}{\text{arg}\;\text{max}}\;s\left(r,R\right). \end{array}$$ (2)

**Fig 3:**
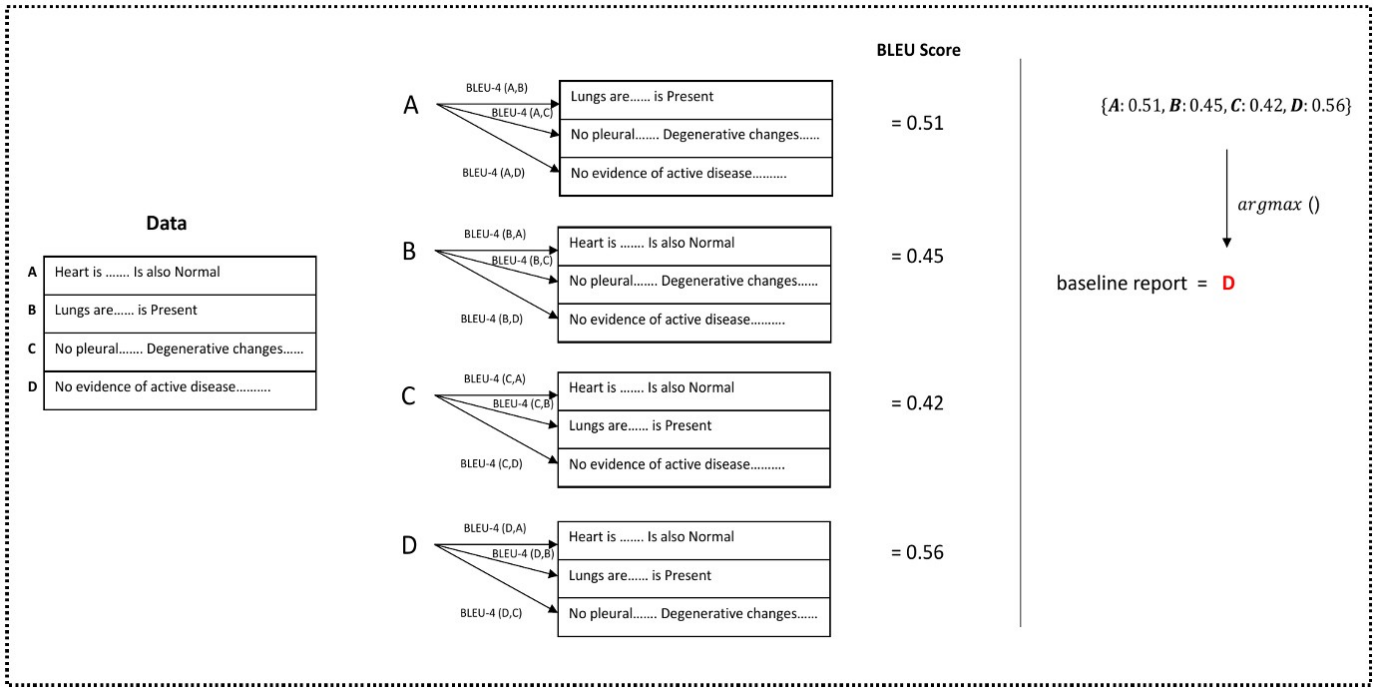
Illustration of unconditioned Baseline 1, which optimizes the BLEU-4 score.

In case more than one report satisfies the above condition, we randomly select one of them. Fig 3 illustrates an application of such a baseline. Note that this baseline method just retrieves a report from the training data.

While this baseline method can be used with any evaluation metric, computing the metric for each report in the training set can still be very expensive. A naive search over a training set of size *N* takes *O*(*N*^2^) time.

Nevertheless, for some metrics, the computation can be sped up.

In particular, for the BLEU scores, we can build a table of the number of times any *K*-gram occurs in the dataset. With this table, the score BLEU- *K*(*c*, *R*) can be computed in time proportional to the length of the report *c*. And this makes it feasible to try all reports in the training set.

For CIDEr and CIDEr-D a similar table can be used to speed up the search, this time multiplying the counts with normalized tf-idf weights. Since CIDEr-D includes a penalty based on the difference in report length, we separate this computation for each possible length. This is still significantly more efficient than a brute force search.

For CheXpert accuracy, we can pre-compute the CheXpert labels for all reports, and count the number of positives and negatives. That makes it possible to calculate the CheXpert accuracy for a candidate report in constant time.

The other NLP metrics (METEOR and ROUGE) are based on sequence matching, which can not be computed more efficiently. For this reason, we use Baseline 1 with BLEU-4, CIDEr-D and CheXpert accuracy in our experiments. We do not consider other BLEU scores or CIDEr, because they are very similar to BLEU-4 and CIDEr, respectively.

**Baseline 2**. For some metrics it is possible to construct the optimal single-report unconditioned model. In particular, for the BLEU-1 score words can be selected independently to form the optimal report. BLEU-1 is a combination of word-level recall, i.e., how many of the words in a reference report are also in a candidate report; and a penalty for short reports. To find a set of words that maximize recall, in the first step of the algorithm we simply rank all words by how often they occur in reports of the training set. The most frequent words are selected, where very frequent words can be selected multiple times. We set the number of selected words to be equal to the average length of the reports in the training set, to avoid a brevity penalty of the BLEU-*k* metric (see section Evaluation Metrics).

We can combine words in any order without changing the BLEU-1 score. Therefore we can attempt to optimize BLEU-2 score as well when combining words to make a report. We do this by greedily joining words into fragments and the fragments into larger fragments, along the most frequent 2-grams. This is an approximate optimization of the precision for 2-grams which, combined with the good set of selected words, heuristically optimizes BLEU-2. Note that reports generated by Baseline 2 may be semantically and grammatically incorrect.

**Fig 4:**
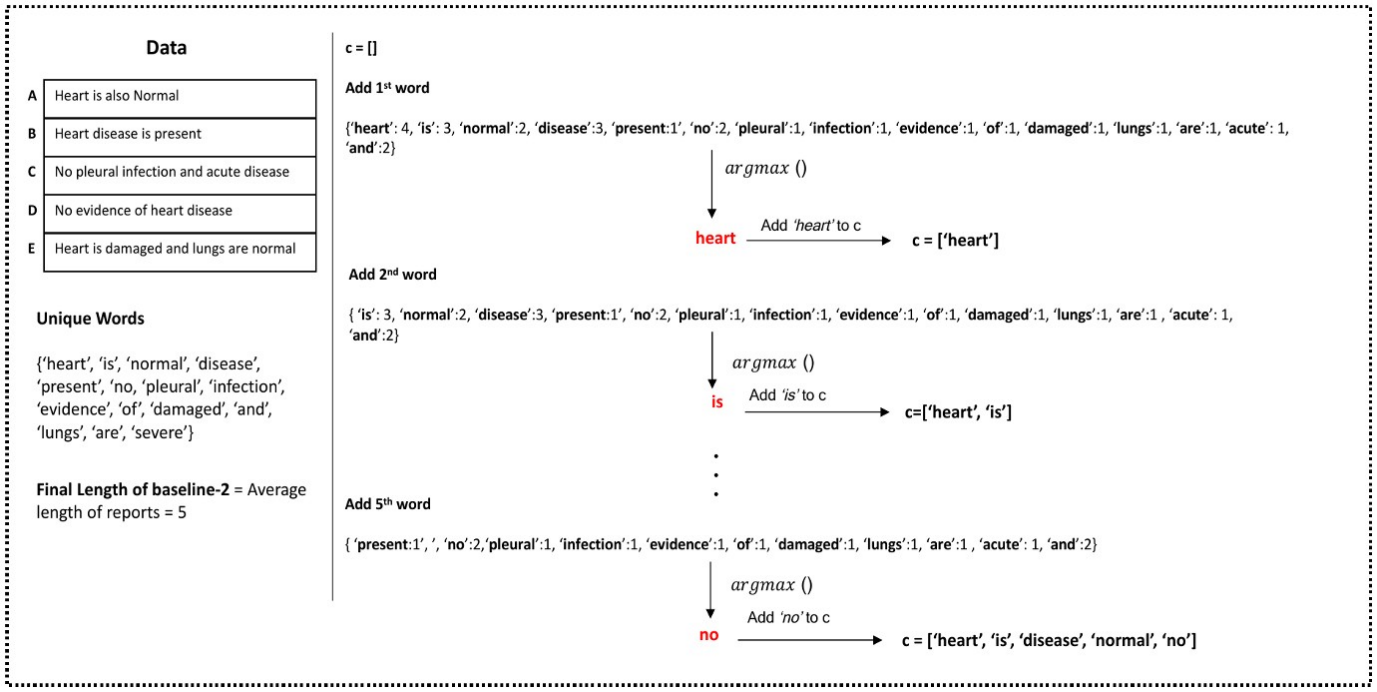
Illustration of unconditioned Baseline 2, which optimizes BLEU-1 and BLEU-2.

These two steps together form our second baseline, see Algorithm 1. Fig 4 illustrates an application of this baseline.

**Algorithm 1**: Baseline 2.

 **Input**: Set *R* of training reports

 **Output**: An approximation *c* of $c_{\text{BLEU}-2}^{*}$

1 Step 1: Set of most frequent words (optimize BLEU-1)

2 *n* ← (∑_*r*∈*R*_ |*r*|)/|*R*|

3 *c* ← []

4 **while** |*c*| < *n* **do**

5  *w* ← argmax_*w*_#{*occ*(*w*, *r*) > *occ*(*w*, *c*) | *r* ∈ *R*}

6  add *w* to *c*

7 **end**

8 Step 2: Join words into larger fragments (optimize BLEU-2)

9 **while** |*c*| > 1 **do**

10  (*a*, *b*) ← next most frequent 2-gram in *R*

11  **if** *find fragments c*_1_ = […, *a*] ∈ *c and c*_2_ = [*b*, …] ∈ *c* **then**

12   remove *c*_1_ and *c*_2_ from *c*

13   add concat(*c*_1_, *c*_2_) to *c*

14  **end**

15 **end**

### Unconditioned permutation models

In addition to the two baselines models, we further investigate the performance of an encoder-decoder model by unconditioning the reports it generates from a test set of X-ray images. We randomly permute the reports generated by an encoder-decoder model *M* applied to a test set (*x*_1_, …, *x*_*m*_) and re-compute the metric scores. We call this procedure *M*-Permuted. In this way, the generated report *c*_*i*_ does not depend anymore on the X-ray input image *x*_*i*_, but rather on another image *x*_*p*(*i*)_, where *p* is a permutation of (1, …, *m*).

Obviously, if the encoder-decoder model *M* was able to incorporate useful information from the image *x*_*i*_ into the generated report *c*_*i*_, then we would expect the performance of *M*-Permuted to be worse. However, if the metric scores of *M* and *M*-Permuted are largely similar, then we can conclude that the model *M* is no better than an unconditioned model.

### Analysis of normal and abnormal cases

In order to investigate the performance of our models on normal and abnormal cases separately, we need to define ‘normal’ and ‘abnormal’. To this aim, we use the external diagnostic tags of reports included in the IU chest X-ray data and split the reports into those with tag ‘normal’, and the rest, which are considered ‘abnormal’. Unfortunately, for the MIMIC-CXR dataset, we cannot perform such an analysis because diagnostic tags are not available.

### Evaluation metrics

The quality of generated reports is in general assessed using standard NLP validation measures: BLEU, ROUGE, METEOR, and CIDEr-D.

In our experiments, we compute these metrics with the Microsoft COCO Caption Evaluation package, which is available at https://github.com/tylin/coco-caption/tree/master/pycocoevalcap. Below, we briefly describe the measures used in our experimental analysis.

Here, let *C* = {*c*_1_, …, *c*_*N*_} denote the set of candidate reports generated by a model when applied to images *x*_1_, …, *x*_*N*_. In the case of an unconditioned model, *c*_*i*_ = *c* for all inputs *x*_*i*_. Let $n_{C}=\sum_{i=1}^{N}\left|c_{i}\right|$ denote the total length of all candidate reports in *C*.

**BLEU**-*K* (BiLingual Evaluation Understudy) [14] is a parametric precision-based metric, with parameter *K*. BLEU-*K* considers sequences of up to *K* words (*K*-grams) in the generated report that occur in the ground-truth one. This metric includes a penalty term for short reports. The BLEU-*K* score of the set *C* of candidate reports with respect to the corresponding set *R* of reference reports is defined as

$$\begin{array}{c} \text{BLEU-}K\left(C,R\right)=BP\left(C,R\right)\cdot \sum_{k=1}^{K}{\text{precision}}_{k}\left(C,R\right), \end{array}$$

where precision_*k*_(*C*, *R*) is the *k*-gram precision between reports in *C* and corresponding reference reports in *R*,

$$\begin{array}{c} {\text{precision}}_{k}\left(C\right)=\frac{1}{n_{C}}\sum_{i=1}^{N}\sum_{w\in W^{k}}\text{min}\left(\text{occ}\left(w,r_{i}\right),\text{occ}\left(w,c_{i}\right)\right). \end{array}$$

Here occ(*w*, *r*) denotes the number of times a *k*-gram *w* occurs in a report *r*. *BP*(*C*) = min(1, exp(1 − *n*_*R*_/*n*_*C*_)) is a brevity penalty term. If *n*_*C*_ ≥ *n*_*R*_, that is, if the average length of candidate reports is longer than that of reference reports, then *BP*(*C*) = 1. If the candidate reports are shorter, then the brevity penalty becomes larger.

**ROUGE** (Recall-Oriented Understudy for Gisting Evaluation) [15] is a family of recall metrics of which the most used is ROUGE-L, an F-score metric defined as follows. For a pair (*c*_*i*_, *r*_*i*_) of candidate and reference reports,

$$\begin{array}{c} \text{ROUGE-L}\left(c_{i},r_{i}\right)=\left(1+\beta^{2}\right)\cdot \frac{\text{LCS}\left(r_{i},c_{i}\right)/|c_{i}|\cdot \text{LCS}\left(r_{i},c_{i}\right)/\left|r_{i}\right|}{\beta^{2}\cdot \text{LCS}\left(r_{i},c_{i}\right)/|c_{i}|+\text{LCS}\left(r_{i},c_{i}\right)/\left|r_{i}\right|}. \end{array}$$

Here LCS(*r*_*i*_, *c*_*i*_) denotes the length of the longest common subsequence of *r*_*i*_ and *c*_*i*_. The parameter *β* is typically set to 1.2. Note that LCS(*r*_*i*_, *c*_*i*_)/|*c*_*i*_| is a kind precision term, measuring how many consecutive words of the candidate appear in the reference, and LCS(*r*_*i*_, *c*_*i*_)/|*r*_*i*_| is a kind of recall. ROUGE-L(*C*, *R*) is defined as the average of the ROUGE- L(*c*_*i*_, *r*_*i*_)’s values.

**METEOR** (Metric for Evaluation of Translation with Explicit ORdering) [16] is defined, for a pair (*c*_*i*_, *r*_*i*_), as the harmonic mean of precision and recall of unigrams matches between *c*_*i*_ and *r*_*i*_, with recall weighted more than precision:

$$\begin{array}{c} \text{METEOR}\left(c_{i},r_{i}\right)=\left(1-0.5\cdot \text{CP}{\left(c_{i},r_{i}\right)}^{3}\right)\cdot \frac{10\cdot \text{precision}\left(c_{i},r_{i}\right)\cdot \text{recall}\left(c_{i},r_{i}\right)}{9\cdot \text{precision}\left(c_{i},r_{i}\right)+\text{recall}\left(c_{i},r_{i}\right)}. \end{array}$$

The penalty term CP(*c*_*i*_, *r*_*i*_) = (number of chunks)/(number of matched unigrams) counts how many contiguous chunks are needed to cover all words that occur in both candidate and reference report. If *c*_*i*_ and *r*_*i*_ are equal then 1 chunk is needed, while if there are gaps then more chunks are needed. METEOR(*C*, *R*) is the average of the METEOR(*c*_*i*_, *r*_*i*_)’s values.

**CIDEr** (Consensus-based Image Description Evaluation) [21] is a more recent metric, defined, for the pair (*c*_*i*_, *r*_*i*_), as

$$\begin{array}{c} {\text{CIDEr}}_{K}\left(c_{i},r_{i}\right)=\frac{1}{K}\sum_{k=1}^{K}\frac{g^{k}\left(c_{i}\right)\cdot g^{k}\left(r_{i}\right)}{\left\|g^{k}\left(c_{i}\right)\right\|\left\|g^{k}\left(r_{i}\right)\right\|}, \end{array}$$

where *g*^*k*^(*c*_*i*_) and *g*^*k*^(*r*_*i*_) denotes *TF-IDF* weighted vectors corresponding to all *k*-grams in *c*_*i*_ and *r*_*i*_, respectively, and ‖*g*^*k*^(*c*_*i*_)‖ and ‖*g*^*k*^(*r*_*i*_)‖ denote their magnitude. The CIDEr-D variant of this metric includes a multiplicative penalty term based on the difference in report length, 10 exp(−(|*c*_*i*_| − |*r*_*i*_|)/2*σ*^2^). Secondly, this variant replaces the inner product *g*^*k*^(*c*_*i*_) ⋅ *g*^*k*^(*r*_*i*_) with min(*g*^*k*^(*c*_*i*_), *g*^*k*^(*r*_*i*_)) ⋅ *g*^*k*^(*r*_*i*_), which means that words that occur more often in the candidate report than in the reference are ignored.

Confusingly, many implementations of CIDEr-D call this metric CIDEr, so it is often unclear which variant was used in published results.

#### CheXpert accuracy

The CheXpert labeleler (available at https://github.com/stanfordmlgroup/chexpert-labeler) is a NLP tool for extracting observations from radiology reports [18] which includes a rule-based model with 14 classes. The labeler is applied to both the ground truth and the candidate reports. Then the accuracy is computed over the extracted labels and used as a diagnostic assessment metric.

### Statistical analysis

We employ a statistical test for machine translation evaluation based on the bootstrap method [22, 23]. As the test statistic we use the difference in scores *δs* = *s*(*M*) − *s*(*M*′) for two methods *M* and *M*′. For example, *s* could be BLEU-1, *M* SA&T, and *M*′ Baseline 2. Under the null-hypothesis that the two methods perform equally well, this statistic has expected value 0. We construct bootstrapped test sets by sampling with replacement from the original test set. The distribution of the test statistic is then estimated by calculating *δs* for each bootstrap sample, which gives a distribution *P*_boot_(*δs*). The distribution of *δs* under the null-hypothesis is derived from the bootstrap distribution, by assuming that it has the same shape but a different mean. The *p*-value is then the probability under the null-hypothesis of the observed *δs* or a more extreme value.

## Results

We have performed a quantitative, qualitative and statistical comparative analysis of the performances of the implemented encoder-decoder models and unconditioned baselines.

In all tables of results, the best values are highlighted in bold face, and ‘-’ indicates that a value is not available.

### IU chest X-ray dataset

On the IU Chest X-Ray dataset, we trained SA&T and MRA, using as encoder the pre-trained deep CNN VGG-19, and cross entropy as the loss function. We performed 60 epochs of the training using a batch size of 16. We used 10^−3^ as the initial learning rate, which was decreased to 10^−4^ after 30 epochs. We trained CDGPT2 with the default parameters given with the code. However, we used a batch size of 4 (this is the maximum we could fit into our GPU’s memory) and the single learning rate of 10^−4^. The Adam optimizer was used to update the weights. For all the methods, we used the same experimental setting, as in [2]. Out of 2775 records, we randomly selected 250 samples to form the test set and used the remaining data for training. We repeated the split of the data into training and test set 5 times, and averaged the results over the test sets.

Also, in order to be able to compare the performance of the considered algorithms with more published methods, we have reported results from the literature for other machine learning methods. From [8] we included the results of the three methods introduced in [8]: **NLG**, which optimizes CIDEr score; **CCR**, which uses reinforcement learning and optimizes clinical accuracy computed using CheXpert; and **Full** which optimizes both natural language coherence and clinical accuracy, **TieNet** [24], an end to end CNN-RNN based ML method performing automatic extraction of distinctive image and text representations, and the traditional image caption methods **S&T** [3] and **SA&T** [4]. From [12], we included results of **VSGRU**, a variation for CDGPT2 which used a Gated Recurrent Unit (GRU) network instead of gpt2. We have also reported results for two other methods used in [12]: **LRCN** [25], a sequential CNN coupled with LSTM; and **ATT-RK** [26], an encoder- decoder model with semantic attention. All the results reported in [8], used only the ‘Findings’ section of the reports, while in [12], all the results were computed using ‘Impression + Findings’.

We conducted two experiments: in the first experiment we used only the ‘Findings’ section of the reports, in order to compare with the recent results from [8] based on this setting; while in the second experiment we used the entire report, that is, both ‘Findings’ and ‘Impression’, since ‘Impression’ is also needed in order to train MRA. The results are reported in Tables 1 and 2. The standard deviations over the multiple runs of the implemented methods are all very small, in the range [0.01, 0.05].

**Table 1:** Average performance results on the IU chest X-ray dataset over test sets.

	BLEU-1	BLEU-2	BLEU-3	BLEU-4	METEOR	ROUGE	CIDEr-D	Accuracy
S&T* [3]	0.265	0.157	0.105	0.073	-	0.306	0.926	-
SA&T* [4]	0.328	0.195	0.123	0.080	-	0.313	1.276	-
TieNet* [24]	0.330	0.194	0.124	0.080	-	0.311	1.334	-
Liu et al. NLG* [8]	0.369	0.246	0.171	0.115	-	**0.359**	**1.490**	-
Liu et al. CCR* [8]	0.160	0.080	0.050	0.036	-	0.244	0.707	-
Liu et al. Full* [8]	0.359	0.237	0.164	0.113	-	0.354	1.424	-
SA&T [4]	0.333	0.210	0.143	0.104	0.150	0.281	0.396	0.877
CDGPT2 [12]	0.360	0.217	0.142	0.096	0.174	0.270	0.249	0.860
Baseline 1 (BLEU-4)	0.422	0.263	0.177	**0.124**	0.169	0.299	0.120	0.891
Baseline 1 (CIDEr-D)	0.384	0.230	0.153	0.101	0.165	0.292	0.290	0.868
Baseline 1 (Accuracy)	0.315	0.176	0.107	0.068	0.160	0.243	0.099	**0.913**
Baseline 2 (BLEU-2)	**0.491**	**0.319**	**0.190**	0.105	**0.203**	0.295	0.288	0.787

Only the ‘Findings’ section of reports is considered. For comparison, we include the results reported in [8] at the top of the table, indicated with *.

**Table 2:** Average performance results on the IU chest X-ray dataset over test sets.

	BLEU-1	BLEU-2	BLEU-3	BLEU-4	METEOR	ROUGE	CIDEr-D	Accuracy
LRCN* [25]	0.369	0.229	0.149	0.099	0.155	0.278	0.111	-
ATT-RK* [26]	0.369	0.226	0.151	0.108	0.171	0.323	0.155	-
VSGRU* [12]	0.347	0.221	0.156	0.116	0.150	0.251	**0.413**	-
SA&T [4]	0.358	0.227	0.154	0.105	0.162	0.298	0.330	0.870
MRA [2]	0.322	0.212	0.142	0.094	0.162	0.280	0.080	0.874
CDGPT2 [12]	0.364	0.231	0.156	0.107	0.161	**0.311**	0.324	0.863
Baseline 1 (BLEU-4)	0.437	0.287	0.194	**0.130**	0.183	0.277	0.267	**0.910**
Baseline 1 (CIDEr-D)	0.425	0.265	0.178	0.116	0.184	0.273	0.321	0.868
Baseline 1 (Accuracy)	0.331	0.183	0.110	0.067	0.163	0.231	0.090	**0.910**
Baseline 2 (BLEU-2)	**0.508**	**0.337**	**0.205**	0.115	**0.213**	0.276	0.260	0.743

Here both the ‘Impression’ and ‘Findings’ section of reports are considered. We include the results reported in the literature, marked with *.

Table 1 contains averaged results over the test sets, for the first setting. When optimizing BLEU or CIDEr-D score, Baselines 1 (BLEU-4 and CIDEr-D) and 2 (BLEU-2) outperformed SA&T, except with respect to CIDEr-D. Also, results indicated that Baseline 1 (BLEU-4 and CIDEr-D) and 2 are competitive with the ML methods reported in [8], except with respect to the ROUGE and CIDEr. Unsurprisingly, optimizing CheXpert accuracy instead of BLEU or CIDEr-D score with Baseline 1 resulted in a higher accuracy, but a lower score for all the NLP metrics. Baseline 2 had inferior CheXpert accuracy, due to its focus on the most frequent words, which have little clinical diagnosis content. We could not explain the relatively high CIDEr values reported in [8]. For instance, there is a large discrepancy between our CIDEr results for SA&T and those from [8] (0.396 for our implementation and 1.276 for the previously published result), while results are in agreement with respect to the other metrics.

Table 2 contains the averaged results over the test sets for our second experiment, that is, when using ‘Findings’ and ‘Impression’. Overall these results are very similar to the first setting. Baselines 1 (optimizing BLEU-4 or CIDEr-D) and Baseline 2 obtained the best performance on the NLP metrics, except for the ROUGE and CIDEr, where SA&T and CDGPT2 outperformed the three unconditioned models. For CIDEr-D, Baseline 1 (CIDEr-D), however, performed on par with the SA&T and CDGPT2. This can be explained by the tendency of our unconditioned models to use words that appear in many reports. With respect to the CheXpert accuracy, the best performance was obtained with the unconditioned Baseline 1, closely followed by MRA.

Table 3 contains the results with the unconditioned permutation models. For each of the models we can observe that, even after randomly permuting the order of the generated reports, the results were slightly worse than but still comparable with those computed with the original order of the reports.

**Table 3:** Results on the IU chest X-ray dataset of permuted models.

	BLEU-1	BLEU-2	BLEU-3	BLEU-4	METEOR	ROUGE	CIDEr-D	Accuracy
SA&T-Permuted	0.334	0.208	0.135	0.088	0.152	0.285	**0.290**	0.862
MRA-Permuted	0.318	0.206	0.139	0.092	**0.162**	0.269	0.067	**0.875**
CDGPT2-Permuted	**0.344**	**0.214**	**0.140**	**0.092**	0.148	**0.288**	0.221	0.854

Here both the ‘Impression’ and ‘Findings’ section of reports are considered. Compare Table 2.

We investigated ‘normal’ and ‘abnormal’ classes separately, and the performance of our models on the two classes. Table 4 contains average performance computed separately for ‘abnormal’ and ‘normal’ reports. As expected, better performance was achieved on ‘normal’ reports than on ‘abnormal’ ones, since about 70% of the reports are ‘normal’, and both classes contain many common parts. Unconditioned baselines remained superior or competitive with SA&T, MRA, and CDGPT2 over both ‘normal’ and ‘abnormal’ reports (except with respect to ROUGE and CIDEr-D, for the reasons explained previously).

**Table 4:** Average validation scores for abnormal and normal reports on the IU chest X-ray dataset (Impressions and Findings).

		BLEU-1	BLEU-2	BLEU-3	BLEU-4	METEOR	ROUGE	CIDEr-D	Accuracy
SA&T	normal	0.400	0.260	0.179	0.120	0.201	**0.333**	0.338	0.893
	abnormal	0.254	0.155	0.098	0.062	0.127	0.242	0.152	0.855
MRA	normal	0.320	0.217	0.153	0.107	0.234	0.307	0.032	0.912
	abnormal	0.327	0.203	0.128	0.080	0.154	0.247	0.066	0.853
CDGPT2	normal	0.359	0.228	0.153	0.103	0.160	0.310	0.329	0.863
	abnormal	0.368	0.234	0.158	0.109	0.162	**0.311**	**0.326**	0.862
Baseline 1 (BLEU-4)	normal	0.440	0.290	0.200	**0.132**	0.235	0.302	0.274	**0.924**
	abnormal	0.382	0.245	0.165	**0.112**	0.162	0.262	0.275	**0.905**
Baseline 1 (CIDEr-D)	normal	0.478	0.332	0.232	0.161	0.261	0.327	**0.565**	0.861
	abnormal	0.354	0.207	0.132	0.081	0.154	0.239	0.187	0.864
Baseline 1 (Accuracy)	normal	0.330	0.187	0.109	0.067	0.192	0.262	0.050	**0.924**
	abnormal	0.337	0.173	0.092	0.052	0.140	0.215	0.099	0.901
Baseline 2	normal	**0.484**	**0.334**	**0.215**	0.128	**0.279**	0.302	0.313	0.734
	abnormal	**0.442**	**0.290**	**0.167**	0.091	**0.182**	0.255	0.246	0.748

### MIMIC-CXR dataset

On the MIMIC-CXR dataset, we could not train SA&T, MRA, and CDGPT2, due to our limited computational resources. Nevertheless, due to the large size of this dataset, results reported in the published works are likely to be representative: we chose to report results from [8], since this recent paper also used the CheXpert clinical validation measure for the quality assessment. On this dataset, we applied 10-fold cross validation to assess the performance of our unconditioned baselines.

We conducted experiments using only the ‘Findings’ part of reports, as in [8]. Table 5 contains averaged results over the test sets. The standard deviations over the multiple folds of our unconditioned baselines were all very small, in the range [0.001, 0.005], which was expected, due to the large size of the dataset. Baseline 1 (optimizing BLEU-4) and Baseline 2 outperformed all other methods with respect to the NLP metrics, except for the ROUGE and CIDEr. Baseline 1 (optimizing CIDEr-D) outperformed all others with respect to the CIDEr-D. Nevertheless, it performed on par with respect to the ROUGE metric and was inferior with respect to the rest of the NLP metrics. Baseline 1 (optimizing CheXpert accuracy) achieved the highest accuracy, closely followed by CCR. The superior performance of Baseline 1 (CIDEr-D) with respect to the CIDEr-D metric and of Baseline 1 (Accuracy) or CCR with respect to accuracy can be explain by the fact that these methods directly optimize these specific metrics.

**Table 5:** Average performance results over test sets on MIMIC-CXR dataset using NLP validation metrics.

	BLEU-1	BLEU-2	BLEU-3	BLEU-4	METEOR	ROUGE	CIDEr-D	Accuracy
S&T* [3]	0.307	0.201	0.137	0.093	-	0.300	0.886	0.837
SA&T* [4]	0.318	0.205	0.137	0.093	-	0.288	0.967	0.849
TieNet* [24]	0.332	0.212	0.142	0.095	-	0.296	1.004	0.848
Liu et al. NLG* [8]	0.352	0.223	0.153	0.104	-	**0.307**	**1.153**	0.834
Liu et al. CCR* [8]	0.294	0.190	0.134	0.094	-	0.284	0.956	0.868
Liu et al. Full* [8]	0.313	0.206	0.146	0.103	-	0.206	1.046	0.867
Baseline 1 (BLEU-4)	0.369	0.237	0.162	**0.115**	0.146	0.306	0.093	0.843
Baseline 1 (CIDEr-D)	0.272	0.171	0.123	0.093	0.134	0.293	**0.269**	0.843
Baseline 1 (Accuracy)	0.310	0.181	0.120	0.083	0.126	0.218	0.053	**0.871**
Baseline 2 (BLEU-2)	**0.448**	**0.283**	**0.175**	0.107	**0.170**	0.203	0.120	0.754

For comparison, we include the results reported in [8] at the top of the table, indicated with *.

In summary, on both datasets, Baseline 1 and 2 outperformed the considered encoder-decoder models with respect to most of the NLP validation metrics, except for ROUGE and CIDEr-D. However, on MIMIC-CXR, Baseline 1 (CIDEr-D) performed best only with respect to the CIDEr-D metric.

The lower ROUGE score of our baselines can be explained by the fact that this is a recall based metric, and unconditioned models consist of a single report, hence they have limited coverage of the ground truth reports, and thus low recall. Lower CIDEr-D score of our baselines, except Baseline 1 (CIDEr-D) was also expected, since this metric penalizes *k*-grams occurring in all reports, which obviously happens a lot in single-report unconditioned models. The NLG variant of the method by Liu et al. and Baseline 1 (CIDEr-D) yielded the best CIDEr-D values, because these method optimize this metric. Similarly, on both datasets, Baseline 1 (Accuracy) achieved the highest accuracy results. The CCR and Full methods by Liu et al. also performed well on this metric, because (the CheXpert) accuracy is also directly optimized by those methods. The single-report baselines that optimize BLEU score (Baseline 1 and 2), although very simple, achieved the best BLEU results.

Unfortunately, for the MIMIC-CXR dataset, we could not perform an analysis of ‘normal’ and ‘abnormal’ classes separately because diagnostic tags were not available.

### Qualitative analysis

**Fig 5:**
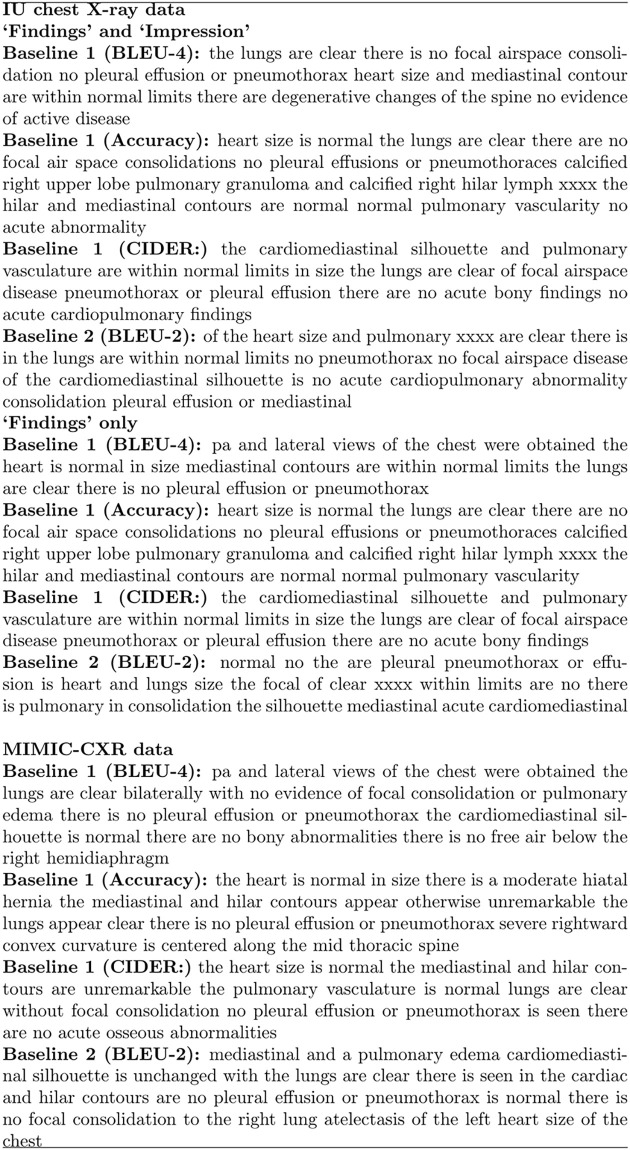
Typical reports generated by Baseline 1 and 2 (over one train/test split).

A qualitative analysis of the results indicated that our unconditioned models are mainly descriptions of the chest X-ray images without abnormal characteristics. Examples of reports that were generated by our unconditioned models are shown in Fig 5. As expected, reports generated by the Baseline 2 are grammatically incorrect and semantically meaningless.

In general, although our unconditioned baselines achieved very good performance, they are not of practical relevance. Indeed, these unconditioned models were developed only as a means for assessing the effectiveness of the encoder-decoder models.

**Fig 6:**
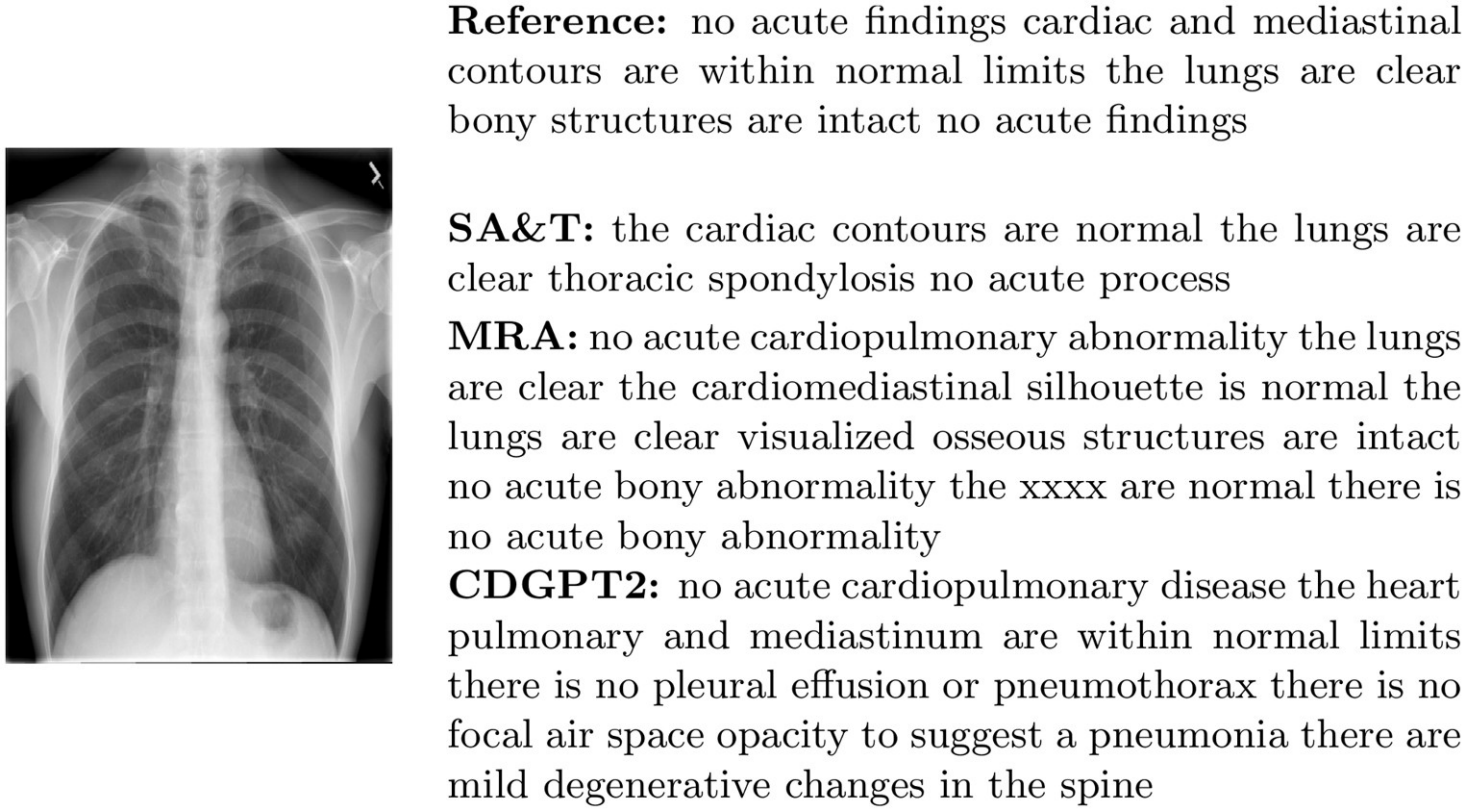
Example of reports generated with encoder-decoder methods. We show SA&T, MRA, and CDGPT2 outputs for an input image in the IU chest X-ray test set, and the reference ground truth report.

**Fig 7:**
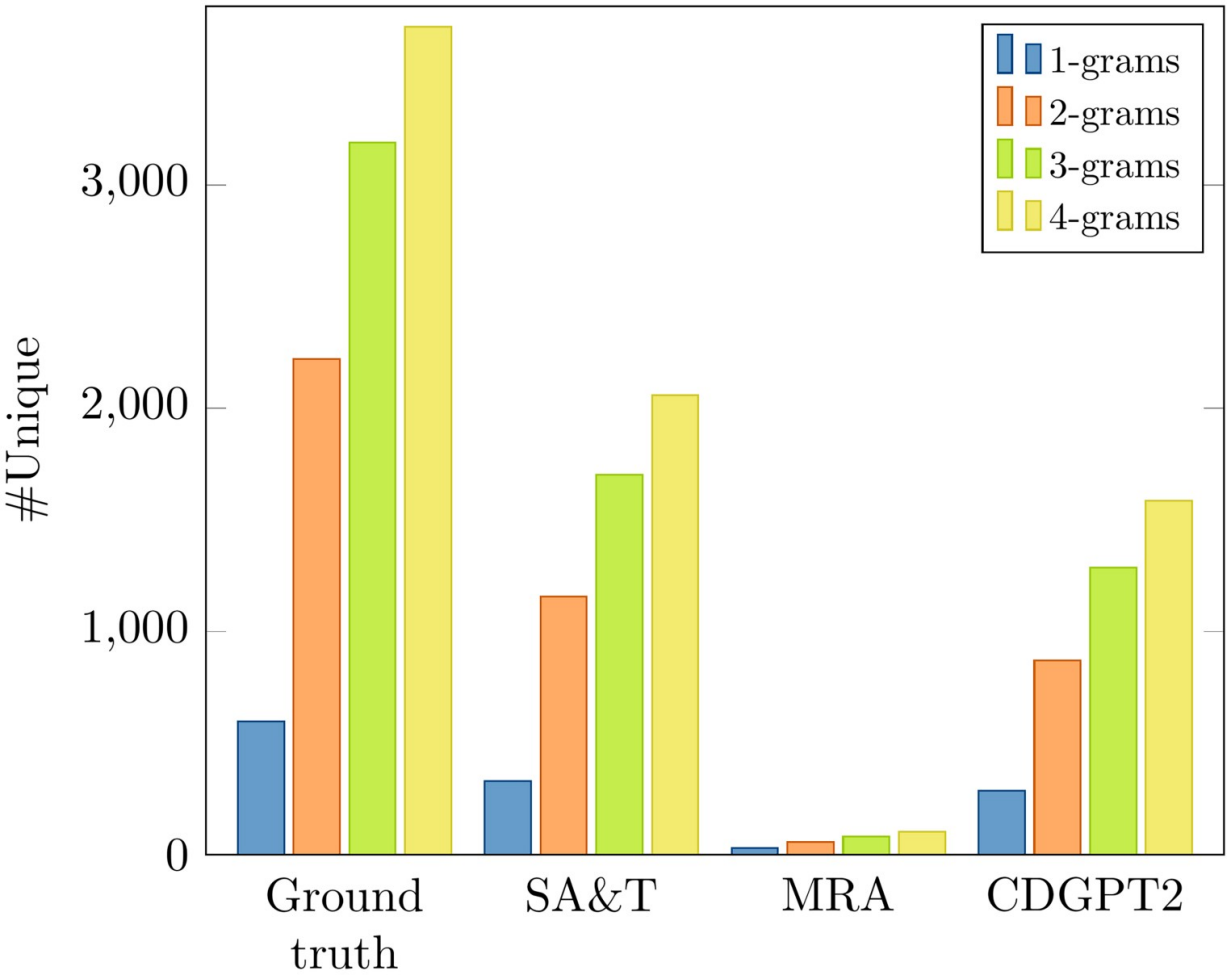
Number of unique n-grams in reports generated by SA&T, MRA, CDGPT2 and in ground truth reports for the IU chest X-ray data. Although reports generated by MRA are longer than those generated by SA&T and CDGPT2, they are more similar to each other. Average length of reports generated by SA&T is 32, by CDGPT2 is 32, and by MRA is 47.

Fig 6 shows an example of a report generated by SA&T, MRA, and by CDGPT2 from a chest image with no abnormal conditions. Overall, reports generated by MRA are less diverse than those generated by SA&T and CDGPT2, as clearly shown in Fig 7. The number of unique n-grams in reports generated by SA&T, MRA, and CDGPT2 is much lower than that in the ground truth reports; reports generated by MRA have a rather low number of unique words.

### Statistical analysis

We performed a statistical analysis of our baselines, SA&T, MRA, and CDGPT2 on the IU chest X-ray dataset (using Impressions and Findings) to assess whether their differences in performance are significance.

**Fig 8:**
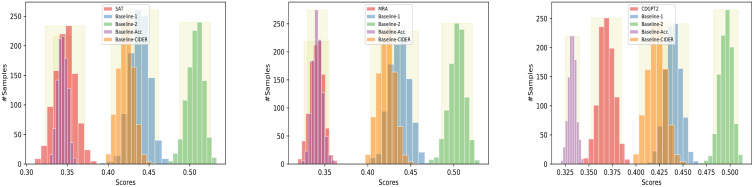
Bootstrap distributions of BLEU-1 values for various pairs of methods. 95% confidence intervals are shown in light yellow color.

In our experiments, we considered 1000 bootstrap samples. Fig 8 shows the bootstrapping frequency distributions of *s*(*M*) and *s*(*M*′) for *s* = BLEU-1 and the 95% confidence intervals, for various pairs of methods. Table A1 in S1 File, contains results obtained by applying the two-sided bootstrapping resampling statistical test to pairs of the methods with respect to the BLEU-1 metric. Results showed that BLEU-1 values of the Baseline 1 (all variants) and 2 were significantly better (*p*-value < 0.05) than those of the SA&T, MRA, and CDGPT2. However, a different behavior was observed with respect to the ROUGE and CIDER metric. In case of the ROUGE (Table A7 in S1 File), S&AT and CDGPT2 performed similarly yet better (*p*-value < 0.05) than the Baselines 1 (all variants) and 2, while there was no significant difference between the performance of MRA and the baseline models (*p*-value > 0.05). In case of the CIDER metric (Table A8 in S1 File), the performance of SAT and CDGPT2 was not significantly different than that of Baselines 1 (BLEU-4 and CIDEr-D variants) and 2 (*p*-value > 0.05), while it was significantly better than that of Baseline-1 (Accuracy). Baseline 1 and 2 performed significantly better than MRA (Table A8 in S1 File). Also, there was no significant difference (*p*-value > 0.05) between the performances of permuted methods and their unpermuted counterparts with respect to all metrics, except ROUGE, where the CDGPT2 was significantly better than CDGPT2-Permuted (*p* < 0.05) (see also Tables A2-A6 in S1 File).

## Discussion

Results of our experiments indicate that the considered encoder-decoder models are not effective, since they were in many cases outperformed by the (simple) unconditioned models. We provided further arguments to substantiate this finding, and showed that SA&T, MRA, and CDGPT2 tended to learn an unconditioned model. Reports generated by SA&T, MRA, and CDGPT2 were rather similar to each other: as shown in Fig 7, the number of unique n-grams in the set of generated reports is much lower than that in the set of the ground truth reports. Also, it takes about 28 unique unigrams, 35 bigrams or only 4 unique sentences to generate reports for the whole corpus. This seemed to suggest that, in a way, SA&T, MRA, and CDGPT2 learn an unconditioned model. This claim was also demonstrated by the permutation experiment and further substantiated by the bootstrap re-sampling based analysis. This analysis indicated no significant difference in performance between the models and their permuted variants with respect to the majority of metrics. These results suggest that encoder-decoder models obtained using SA&T, MRA, or CDGPT2 do not make effective use of the (content of the) input image.

### Downsides of the medical report structure

In order to understand why SA&T, MRA, and CDGPT2 tend to learn an unconditioned model, we looked at the structure and content of the medical reports. A medical report of a chest X-ray image mainly contains observations related to the four major types of parameters: 1) The size and outline of the heart 2) The condition of the lungs 3) Heart-related lung problems 4) Fractures in bony structures. It is quite rare that any of the reports leave out observations regarding heart, lungs, bony structures, or even relevant diseases. That is, reports are rather standardized and overlapping. Also, it is very unlikely that a report contains observations concerning abnormalities in each of the above four topics. These observations explain the relatively good performance achieved by a single ‘good’ report that does not refer to the abnormal conditions.

One could conjecture that encoder-decoder ML methods that optimize diagnostic scores, like accuracy computed using the CheXpert labeler tool, could achieve better performance. Results of our investigation show that this is not the case, since results indicate that even Full [8] is outperformed by our unconditioned baselines. Also, as shown e.g. in [8], even when using accuracy computed using the CheXpert labeler as the validation metric, results become only marginally better than those of the majority class classifier that always predicts negative findings. Our investigation provides a more in-depth analysis of the effectiveness of encoder-decoder based models: unconditioned models can outperform encoder-decoder models even when diagnostic validation metrics are used, and encoder-decoder models tend to be unconditioned.

## Conclusion

In summary, we have presented a framework based on the unconditioned models to investigate the effectiveness of the encoder-decoder models for generating the radiology reports from the chest X-ray images. We have shown experimentally that simple unconditioned models are competitive with the encoder-decoder models. Results of our investigation indicated that intrinsic characteristics of the radiology reports drive the encoder-decoder models to learn the descriptions commonly used in both the normal and abnormal reports, that is, encoder-decoder models tend to learn unconditioned models.

A limitation of our contribution is that, although we have demonstrated the ineffectiveness of three encoder-decoder models, we do not provide a way to make encoder-decoder models more effective. A promising approach in this direction, employed in recent methods, is the use of prior knowledge in the form of diagnostic labels, in order to guide a method to learn (or retrieve) a more informative report, see e.g., [7, 27, 28]. For instance, in [7] the authors propose to separate abnormal and normal sentence generation. Each sentence of a report is annotated with a label (abnormal or normal). The labeled dataset is then used to train a new model combined with an abnormal sentence predictor. To this aim, MTI encodings keywords are automatically extracted from the indication and findings part of a report. A set of the unique MTI labels in the dataset are identified and used for an additional training signal (the abnormal sentence predictor). In [28] a set of core findings label vocabulary was derived through a multi-year chest X-ray lexicon building effort involving several radiologists and clinical experts, and were used to associate labels to radiology reports using natural language analysis. The resulting labels were used to train a deep learning network to predict finding labels. For a new chest X-ray image, the predicted finding labels are matched against a pre-assembled database of label patterns and their associated reports, in order to retrieve a best report for that image. The retrieved report is then post-processed to remove mentioned findings whose evidence is absent in the predicted label pattern. Although promising, these methods use prior information in the form of labels associated to reports, and such information may not be available or be difficult to obtain. Future work involves an extension of our framework to assess the effectiveness of more involved encoder-decoder methods, like those above mentioned.

A general aspect of radiology report generation that needs a more in-depth analysis is the clinical perspective. In this paper, as in some previous works on machine learning for radiology report generation, the clinical perspective is only implicitly addressed through the use of a diagnostic evaluation metrics like diagnostic accuracy, which quantify the diagnostic content of generated reports. To be clinically relevant, the generated report should not only accurately describe findings in the image, but also trigger the correct (re)action of the clinician. How to model and assess this important issue is in our opinion an interesting open problem for future research.

## Supporting information

S1 File Results of the bootstrap resampling significance test (with 1000 bootstrap samples) applied to all the NLP validation score metrics. (PDF)
