# Supplementary material for: Encoder-decoder models for chest X-ray report generation perform no better than unconditioned baselines
Source: PLoS One. 2021 Nov 29;16(11):e0259639. doi: 10.1371/journal.pone.0259639 (PMC8629217; doi:10.1371/journal.pone.0259639)
Supplement: S1 File — (PDF) [file pone.0259639.s001.pdf]

S1 File

November 16, 2021

Table A1: **Results of the bootstrap resampling significance test (with 1000 bootstrap samples) applied to BLEU-1 scores.**

**Methods**	**95% Confidence Intervals**	**means $\bar{s}$**	**p-value**
SA&T, Baseline 1 (BLEU-4)	[0.323, 0.371], [0.420, 0.461]	0.347, 0.438	<.001
SA&T, Baseline 1 (CIDER)	[0.323, 0.371], [0.404, 0.439]	0.347, 0.422	<.001
SA&T, Baseline 1 (Accuracy)	[0.323, 0.371], [0.333, 0.355]	0.347, 0.344	>0.05
SA&T, Baseline 2 (BLEU-2)	[0.323, 0.371], [0.485, 0.523]	0.347, 0.506	<.001
SA&T, SA&T-Permuted	[0.323, 0.371], [0.321, 0.370]	0.347, 0.346	>0.05
MRA, Baseline 1 (BLEU-4)	[0.326, 0.354], [0.415, 0.458]	0.340, 0.437	<.001
MRA, Baseline 1 (CIDER)	[0.326, 0.354], [0.404, 0.439]	0.340, 0.422	<.001
MRA, Baseline 1 (Accuracy)	[0.326, 0.354], [0.330, 0.354]	0.340, 0.341	>0.05
MRA, Baseline 2 (BLEU-2)	[0.326, 0.354], [0.484, 0.520]	0.340, 0.503	<.001
MRA, MRA-Permuted	[0.326, 0.354], [0.324, 0.354]	0.340, 0.340	>0.05
CDGPT2, Baseline 1 (BLEU-4)	[0.353, 0.384], [0.423, 0.455]	0.368, 0.439	<.001
CDGPT2, Baseline 1 (CIDER)	[0.353, 0.384], [0.404, 0.440]	0.368, 0.422	<.001
CDGPT2, Baseline 1 (Accuracy)	[0.353, 0.384], [0.324, 0.340]	0.368, 0.332	<.002
CDGPT2, Baseline 2 (BLEU-2)	[0.353, 0.384], [0.481, 0.510]	0.368, 0.495	<.001
CDGPT2, CDGPT2-Permuted	[0.353, 0.384], [0.337, 0.370]	0.368, 0.354	>0.05

Table A2: **Results of the bootstrap resampling significance test (with 1000 bootstrap samples) applied to METEOR scores.**

**Methods**	**95% Confidence Intervals**	**means $\bar{s}$**	**p-value**
SA&T, Baseline 1 (BLEU-4)	[0.151, 0.170], [0.175, 0.194]	0.160, 0.184	<.004
SA&T, Baseline 1 (CIDER)	[0.151, 0.170], [0.177, 0.193]	0.160, 0.185	<.004
SA&T, Baseline 1 (Accuracy)	[0.151, 0.170], [0.159, 0.175]	0.160, 0.166	>0.05
SA&T, Baseline 2 (BLEU-2)	[0.151, 0.170], [0.204, 0.224]	0.160, 0.214	<.001
SA&T, SA&T-Permuted	[0.151, 0.170], [0.151, 0.170]	0.160, 0.160	>0.05
MRA, Baseline 1 (BLEU-4)	[0.173, 0.192], [0.174, 0.193]	0.183, 0.183	<.001
MRA, Baseline 1 (CIDER)	[0.173, 0.192], [0.177, 0.193]	0.183, 0.185	<.001
MRA, Baseline 1 (Accuracy)	[0.173, 0.192], [0.158, 0.176]	0.183, 0.166	>0.05
MRA, Baseline 2 (BLEU-2)	[0.173, 0.192], [0.203, 0.223]	0.183, 0.212	<.001
MRA, MRA-Permuted	[0.173, 0.192], [0.171, 0.192]	0.183, 0.181	>0.05
CDGPT2, Baseline 1 (BLEU-4)	[0.152, 0.167], [0.178, 0.191]	0.159, 0.184	<.001
CDGPT2, Baseline 1 (CIDER))	[0.152, 0.167], [0.177, 0.193]]	0.159, 0.185	<.001
CDGPT2, Baseline 1 (Accuracy)	[0.152, 0.167], [0.155, 0.167]	0.159, 0.161	>0.05
CDGPT2, Baseline 2 (BLEU-2)	[0.152, 0.167], [0.200, 0.216]	0.159, 0.210	<.001
CDGPT2, CDGPT2-Permuted	[0.152, 0.167], [0.145, 0.159]	0.159, 0.152	>0.05

Table A3: **Results of the bootstrap resampling significance test (with 1000 bootstrap samples) applied to CheXpert based accuracy scores.**

**Methods**	**95% Confidence Intervals**	**means $\bar{s}$**	**p-value**
SA&T, Baseline 1 (BLEU-4)	[0.844, 0.874], [0.897, 0.914]	0.860, 0.906	<.001
SA&T, Baseline 1 (CIDER)	[0.844, 0.874], [0.854, 0.867]	0.860, 0.861	>0.05
SA&T, Baseline 1 (Accuracy)	[0.844, 0.874], [0.898, 0.913]	0.860, 0.906	<.001
SA&T, Baseline 2 (BLEU-2)	[0.844, 0.874], [0.737, 0.754]	0.860, 0.745	<.001
SA&T, SA&T-Permuted	[0.844, 0.874], [0.846, 0.871]	0.860, 0.860	>0.05
MRA, Baseline 1 (BLEU-4)	[0.859, 0.885], [0.895, 0.911]	0.873, 0.904	<.001
MRA, Baseline 1 (CIDER)	[0.859, 0.885], [0.854, 0.867]	0.873, 0.861	>0.05
MRA, Baseline 1 (Accuracy)	[0.859, 0.885], [0.895, 0.912]	0.873, 0.904	<.001
MRA, Baseline 2 (BLEU-2)	[0.859, 0.885], [0.736, 0.753]	0.873, 0.744	<.001
MRA, MRA-Permuted	[0.859, 0.885], [0.858, 0.884]	0.873, 0.872	>0.05
CDGPT2, Baseline 1 (BLEU-4)	[0.851, 0.867], [0.897, 0.909]	0.859, 0.903	<.001
CDGPT2, Baseline 1 (CIDER)	[0.851, 0.867], [0.854, 0.867]	0.859, 0.861	>0.05
CDGPT2, Baseline 1 (Accuracy)	[0.851, 0.867], [0.897, 0.909]	0.859, 0.904	<.001
CDGPT2, Baseline 2 (BLEU-2)	[0.851, 0.867], [0.742, 0.754]	0.859, 0.748	<.001
CDGPT2, CDGPT2-Permuted	[0.851, 0.867], [0.848, 0.864]	0.859, 0.856	>0.05

Table A4: **Results of the bootstrap resampling significance test (with 1000 bootstrap samples) applied to BLEU-2 scores.**

**Methods**	**95% Confidence Intervals**	**means $\bar{s}$**	**p-value**
SA&T, Baseline 1 (BLEU-4)	[0.201, 0.238], [0.270, 0.304]	0.219, 0.287	<.001
SA&T, Baseline 1 (CIDER)	[0.201, 0.238], [0.252, 0.282]	0.219, 0.267	<.002
SA&T, Baseline 1 (Accuracy)	[0.201, 0.238], [0.183, 0.206]	0.219, 0.194	<.001
SA&T, Baseline 2 (BLEU-2)	[0.201, 0.238], [0.322, 0.352]	0.219, 0.337	<.001
SA&T, SA&T-Permuted	[0.201, 0.238], [0.199, 0.238]	0.219, 0.218	>0.05
MRA, Baseline 1 (BLEU-4)	[0.215, 0.238], [0.268, 0.302]	0.227, 0.285	<.001
MRA, Baseline 1 (CIDER)	[0.215, 0.238], [0.252, 0.282]	0.227, 0.267	<.001
MRA, Baseline 1 (Accuracy)	[0.215, 0.238], [0.182, 0.204]	0.227, 0.192	<.001
MRA, Baseline 2 (BLEU-2)	[0.215, 0.238], [0.320, 0.348]	0.227, 0.335	<.001
MRA, MRA-Permuted	[0.215, 0.238], [0.213, 0.236]	0.227, 0.225	>0.05
CDGPT2, Baseline 1 (BLEU-4)	[0.217, 0.243], [0.273, 0.298]	0.230, 0.285	<.001
CDGPT2, Baseline 1 (CIDER)	[0.217, 0.243], [0.252, 0.282]	0.230, 0.267	<.001
CDGPT2, Baseline 1 (Accuracy)	[0.217, 0.243], [0.181, 0.197]	0.230, 0.189	<.001
CDGPT2, Baseline 2 (BLEU-2)	[0.217, 0.243], [0.319, 0.340]	0.230, 0.330	<.001
CDGPT2, CDGPT2-Permuted	[0.217, 0.243], [0.206, 0.233]	0.230, 0.219	>0.05

Table A5: **Results of the bootstrap resampling significance test (with 1000 bootstrap samples) applied to BLEU-3 scores.**

**Methods**	**95% Confidence Intervals**	**means $\bar{s}$**	**p-value**
SA&T, Baseline 1 (BLEU-4)	[0.129, 0.164], [0.181, 0.212]	0.146, 0.196	<.001
SA&T, Baseline 1 (CIDER)	[0.129, 0.164], [0.168, 0.197]	0.146, 0.181	<.029
SA&T, Baseline 1 (Accuracy)	[0.129, 0.164], [0.101, 0.125]	0.146, 0.112	<.001
SA&T, Baseline 2 (BLEU-2)	[0.129, 0.164], [0.193, 0.216]	0.146, 0.205	<.001
SA&T, SA&T-Permuted	[0.129, 0.164], [0.129, 0.163]	0.146, 0.145	>0.05
MRA, Baseline 1 (BLEU-4)	[0.145, 0.166], [0.179, 0.210]	0.156, 0.194	<.001
MRA, Baseline 1 (CIDER)	[0.145, 0.166], [0.168, 0.197]	0.156, 0.181	<.001
MRA, Baseline 1 (Accuracy)	[0.145, 0.166], [0.101, 0.124]	0.156, 0.111	<.001
MRA, Baseline 2 (BLEU-2)	[0.145, 0.166], [0.192, 0.215]	0.156, 0.203	<.001
MRA, MRA-Permuted	[0.145, 0.166], [0.144, 0.164]	0.159, 0.154	>0.05
CDGPT2, Baseline 1 (BLEU-4)	[0.140, 0.162], [0.184, 0.205]	0.150, 0.194	<.001
CDGPT2, Baseline 1 (CIDER)	[0.140, 0.162], [0.168, 0.197]	0.150, 0.181	<.002
CDGPT2, Baseline 1 (Accuracy)	[0.140, 0.162], [0.102, 0.118]	0.150, 0.109	<.001
CDGPT2, Baseline 2 (BLEU-2)	[0.140, 0.162], [0.193, 0.210]	0.150, 0.202	<.001
CDGPT2, CDGPT2-Permuted	[0.140, 0.162], [0.132, 0.155]	0.150, 0.143	>0.05

Table A6: **Results of the bootstrap resampling significance test (with 1000 bootstrap samples) applied to BLEU-4 scores.**

**Methods**	**95% Confidence Intervals**	**means** $\bar{s}$	p**-value**
SA&T, Baseline 1 (BLEU-4)	[0.084, 0.116], [0.119, 0.148]	0.100, 0.133	<.023
SA&T, Baseline 1 (CIDER)	[0.084, 0.116], [0.108, 0.137]	0.100, 0.121	>0.05
SA&T, Baseline 1 (Accuracy)	[0.084, 0.116], [0.060, 0.085]	0.100, 0.069	<.002
SA&T, Baseline 2 (BLEU-2)	[0.084, 0.116], [0.106, 0.124]	0.100, 0.115	>0.05
SA&T, SA&T-Permuted	[0.084, 0.116], [0.084, 0.115]	0.100, 0.098	>0.05
MRA, Baseline 1 (BLEU-4)	[0.095, 0.114], [0.118, 0.147]	0.105, 0.132	<.002
MRA, Baseline 1 (CIDER)	[0.095, 0.114], [0.108, 0.137]	0.105, 0.121	<.020
MRA, Baseline 1 (Accuracy)	[0.095, 0.114], [0.059, 0.083]	0.105, 0.068	<.002
MRA, Baseline 2 (BLEU-2)	[0.095, 0.114], [0.105, 0.123]	0.105, 0.114	<.001
MRA, MRA-Permuted	[0.095, 0.114], [0.094, 0.112]	0.105, 0.103	>0.05
CDGPT2, Baseline 1 (BLEU-4)	[0.090, 0.112], [0.121, 0.141]	0.100, 0.131	<.002
CDGPT2, Baseline 1 (CIDER)	[0.090, 0.112], [0.108, 0.137]	0.100, 0.121	>0.05
CDGPT2, Baseline 1 (Accuracy)	[0.090, 0.112], [0.060, 0.077]	0.100, 0.067	<.001
CDGPT2, Baseline 2 (BLEU-2)	[0.090, 0.112], [0.107, 0.119]	0.100, 0.114	>0.05
CDGPT2, CDGPT2-Permuted	[0.090, 0.112], [0.085, 0.108]	0.100, 0.096	>0.05

Table A7: **Results of the bootstrap resampling significance test (with 1000 bootstrap samples) applied to ROUGE scores.**

**Methods**	**Confidence Intervals (95%)**	**means** $\bar{s}$	p**-value**
SA&T, Baseline 1 (BLEU-4)	[0.287, 0.322], [0.267, 0.290]	0.304, 0.278	<.035
SA&T, Baseline 1 (CIDER)	[0.287, 0.322], [0.258, 0.280]	0.304, 0.269	<.020
SA&T, Baseline 1 (Accuracy)	[0.287, 0.322], [0.225, 0.245]	0.304, 0.235	<.001
SA&T, Baseline 2 (BLEU-2)	[0.287, 0.322], [0.268, 0.286]	0.304, 0.277	<.035
SA&T, SA&T-Permuted	[0.287, 0.322], [0.286, 0.321]	0.304, 0.304	>0.05
MRA, Baseline 1 (BLEU-4)	[0.260, 0.283], [0.265, 0.288]	0.272, 0.276	>0.05
MRA, Baseline 1 (CIDER)	[0.260, 0.283], [0.258, 0.280]]	0.272, 0.269	>0.05
MRA, Baseline 1 (Accuracy)	[0.260, 0.283], [0.225, 0.243]	0.272, 0.234	<.001
MRA, Baseline 2 (BLEU-2)	[0.260, 0.283], [0.266, 0.283]	0.272, 0.275	>0.05
MRA, MRA-Permuted	[0.260, 0.283], [0.258, 0.279]	0.272, 0.268	>0.05
CDGPT2, Baseline 1 (BLEU-4)	[0.290, 0.311], [0.266, 0.282]	0.300, 0.274	<.001
CDGPT2, Baseline 1 (CIDER)	[0.290, 0.311], [0.258, 0.280]	0.300, 0.269	<.001
CDGPT2, Baseline 1 (Accuracy)	[0.290, 0.311], [0.220, 0.234]	0.300, 0.227	<.001
CDGPT2, Baseline 2 (BLEU-2)	[0.290, 0.311], [0.265, 0.277]	0.300, 0.270	<.001
CDGPT2, CDGPT2-Permuted	[0.290, 0.311], [0.275, 0.297]	0.300, 0.286	<.003

Table A8: **Results of the bootstrap resampling significance test (with 1000 bootstrap samples) applied to CIDER scores.**

**Methods**	**Confidence Intervals (95%)**	**means $\bar{s}$**	**p-value**
SA&T, Baseline 1 (BLEU-4)	[0.197, 0.449], [0.267, 0.292]	0.310, 0.273	>0.05
SA&T, Baseline 1 (CIDER)	[0.197, 0.449], [0.257, 0.425]	0.310, 0.336	>0.05
SA&T, Baseline 1 (Accuracy)	[0.197, 0.449], [0.061, 0.211]	0.310, 0.115	<.003
SA&T, Baseline 2 (BLEU-2)	[0.197, 0.449], [0.221, 0.291]	0.310, 0.256	>0.05
SA&T, SA&T-Permuted	[0.197, 0.449], [0.198, 0.438]	0.310, 0.303	>0.05
MRA, Baseline 1 (BLEU-4)	[0.055, 0.112], [0.225, 0.312]	0.081, 0.268	<.001
MRA, Baseline 1 (CIDER)	[0.055, 0.112], [0.257, 0.425]	0.081, 0.336	<.001
MRA, Baseline 1 (Accuracy)	[0.055, 0.112], [0.060, 0.201]	0.081, 0.111	>0.05
MRA, Baseline 2 (BLEU-2)	[0.055, 0.112], [0.218, 0.288]	0.081, 0.252	<.001
MRA, MRA-Permuted	[0.055, 0.112], [0.056, 0.100]	0.081, 0.076	>0.05
CDGPT2, Baseline 1 (BLEU-4)	[0.222, 0.390], [0.232, 0.291]	0.294, 0.261	>0.05
CDGPT2, Baseline 1 (CIDER)	[0.222, 0.390], [0.257, 0.425]	0.294, 0.336	>0.05
CDGPT2, Baseline 1 (Accuracy)	[0.222, 0.390], [0.054, 0.149]	0.294, 0.093	<.001
CDGPT2, Baseline 2 (BLEU-2)	[0.222, 0.390], [0.149, 0.183]	0.294, 0.166	<.001
CDGPT2, CDGPT2-Permuted	[0.222, 0.390], [0.174, 0.311]	0.294, 0.236	>0.05
